# Factors affecting association between pain severity and physical activity among people with low back pain

**DOI:** 10.1097/MRR.0000000000000591

**Published:** 2023-07-07

**Authors:** Mikhail Saltychev, Henri Hellgren, Juhani Juhola

**Affiliations:** a Department of Physical and Rehabilitation Medicine, Turku University Hospital and University of Turku; b Clinical Division, Faculty of Medicine, University of Turku, Turku, Finland

**Keywords:** disability evaluation, exercise, health risk behaviors, low back pain, sedentary behavior

## Abstract

The aim was to identify factors, which may affect the relationship between physical activity and pain severity among patients with low back pain (LBP). It was a cross-sectional survey-based study among 1332 consecutive patients with LBP. Linear regression models were employed. Patients were 47.6 years old and 64% were women. For the entire sample, pain severity and the intensity of physical activity were negatively associated. Higher physical activity was associated with younger age, higher educational level, normal weight and optimal perceived general health. Sex, smoking, marital status and occupation did not demonstrate significant interactions on the association. The severity of disability showed paradoxical effect on the relationship between pain and physical activity – severe disability was associated with increase in physical activity.

## Introduction

The relationship between the intensity of physical activity and the severity of low back pain (LBP) has widely been studied showing inconsistent results [1–3]. This association has usually been found to be negative [1]. It has been suggested that sufficient physical activity might reduce the risk of chronic LBP by up to 16% [3]. Some previous research has been unable to detect a relationship between physical activity and LBP [4]. A few studies have stated that this association might be nonlinear showing a U-shaped relationship – both insufficient and excessive physical activity might be associated with worse LBP [2]. While this inconsistency might be the result of diversity among methods used to grade the amount of physical activity or LBP, it has been suggested that the association between physical activity and LBP might be influenced by the individual characteristics of patients [2,4]. Therefore, such factors as demographics, disability severity or general health status may affect the strength or even the direction of association between physical activity and LBP.

While severe disability due to chronic LBP has been associated with lower physical activity, no such correlation has been seen among people with acute or subacute LBP [5]. Male sex has been seen to be associated with higher physical activity and lower prevalence of LBP [6–8]. Another study has found that male sex, normal BMI, less pain, and milder disability level are associated with higher physical activity in LBP [9]. While the effects of age and educational level on the association between physical activity and LBP have not directly been studied, indirect speculations could be drawn from previous research. For example, LBP intensity is usually growing towards its peak at 60 years, decreasing after that [10]. In turn, low educational status has been associated with a higher prevalence of LBP [11]. Also, some occupational groups have been connected to worse LBP [12] and lower physical activity [13]. The aim of this study was to detect factors, which may affect the relationship between physical activity and pain severity among patients with LBP.

## Methods

This was a cross-sectional survey-based cohort study of consecutive patients suffering from LBP solely or in addition to other musculoskeletal disorders. They were seen in an outpatient Physical and Rehabilitation Medicine university clinic in 2014–2017. The survey included the Oswestry Disability Index (ODI), questions regarding leisure-time physical activity, pain intensity, demographics, educational level and general health. Of 3150 patients, 1332 responded to the survey (response rate 42%). The study protocol was approved by a university hospital ethics committee.

The ODI includes 10 questions assessed on a scale from 0 (‘no limitation’) to 5 (‘worst possible limitation’). The ODI score is the sum of responses divided by 50 and multiplied by 100. A score of 100% represents the lowest level of functioning and total dependence. The ODI is expressed as percentage. Here, the scores were dichotomized as ‘mild or no disability’ (0–29%) versus ‘moderate or severe disability’ (>29%). The physical activity was calculated from the responses to a standardized questionnaire regarding the different types of physical activity during a week, and the results were calculated in MET-h/week. Pain intensity was defined as the worst pain during the last week experienced using a numeric rating scale from 0 to 10 where 0 signifies ‘no pain’ and 10 signifies ‘worst possible pain’. Three equal age groups were formed based on the age at the time of the survey. Educational level was dichotomized as high school yes versus no. Occupational status was defined according to the International Standard Classification of Occupations and combined into three categories: managers, technicians, and manual workers. Body weight was dichotomized based on the BMI as <31 kg/m^2^ versus >30 kg/m^2^. Marital status was dichotomized as single versus cohabiting. Perceived general health was defined using a Likert-like scale from 1 to 5 with 1 indicating ‘best possible health’ and 5 ‘the worst’. The scores were dichotomized as ‘optimal health’ (score ‘1’ or ‘2’) versus ‘suboptimal health’ (scores ‘3’, ‘4’ or ‘5’).

### Statistical analysis

Firstly, it was investigated if the independent variables show statistically significant (*P* < 0.05) interactions with linear regression models of physical activity on LBP severity. Secondly, models with significant interactions were displayed and analyzed graphically. All the data analyses were performed utilizing Stata 17 (College Station, Texas, USA).

## Results

**Fig. 1:**
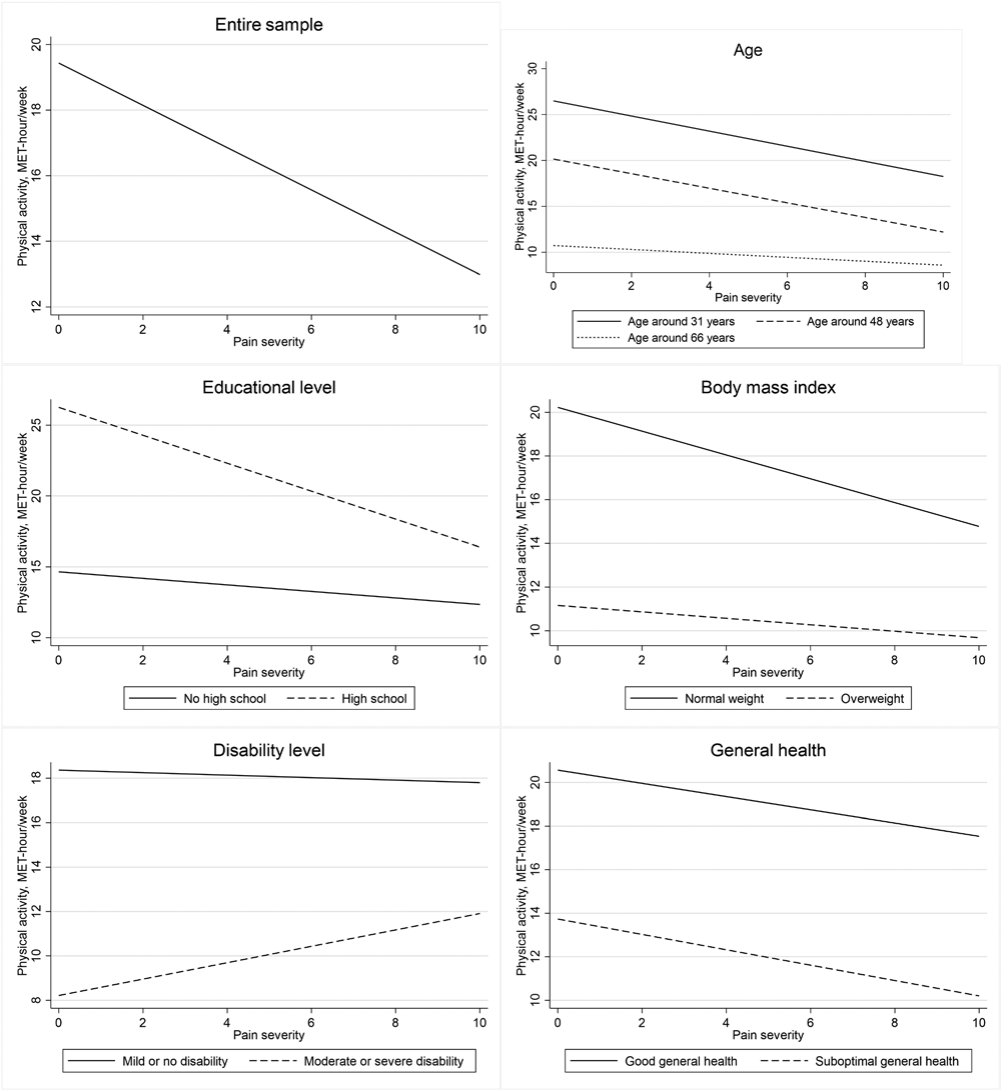
Linear regression of physical activity on pain severity by variables with statistically significant interactions.

The descriptive characteristics are shown in Table 1. For the entire sample, the LBP severity and the intensity of physical activity were negatively associated – the worse was LBP, the less was physical activity (Fig. 1). The same negative association was seen for all the other models (with one exception – people with moderate or severe disability) grouped by the studied independent variables. Younger age, higher educational level, lower BMI and optimal perceived general health were associated with overall higher physical activity. For all these groups, the steepness of linear association between physical activity and LBP severity was similar. Those who reported mild or no disability due to LBP showed high level of physical activity almost apart from pain severity – the regression line was nearly horizontal. Instead, moderate or severe disability showed a paradoxical inverse association – worsening disability was associated with increasing physical activity. Sex, smoking, marital status and occupation did not demonstrate significant interactions on the association between LBP and physical activity.

**Table 1:** Characteristics of the sample and regression coefficients

Variable	*n* (%)	Regression coefficient		
Sex		Slope	95% CI	
Men	482 (36%)	−0.11	−0.84	0.63
Women	850 (64%)	−1.02	−1.59	−0.44
BMI, kg/m^2^^a^				
Normal weight <31 kg/m^2^	956 (73%)	−0.55	−1.09	0.00
Overweight >30 kg/m^2^	356 (27%)	−0.15	−0.94	0.65
Marital				
Single	324 (24%)	−0.77	−1.71	0.17
Co-habiting	1008 (76%)	−0.59	−1.11	−0.08
Educational level^a^				
High school	385 (30%)	−0.23	−0.73	0.27
No high school	888 (70%)	−0.99	−1.94	−0.03
Occupation				
Managers	184 (19%)	0.40	−0.80	1.60
Technicians	258 (27%)	−1.22	−2.29	−0.16
Manual workers	516 (54%)	−0.93	−1.72	−0.15
General health^a^				
Good health	611 (46%)	−0.30	−0.98	0.38
Suboptimal health	721 (54%)	−0.35	−0.91	0.21

Variable	Mean (SD)			
Physical activity, MET-h/week	14.2 (13.8)			
Pain, points	8.2 (1.6)	−0.64	−1.09	−0.19
Age, years^a^	47.6 (15.0)			
Age group 1 *N* = 442	30.7 (7.3)	−0.80	−1.69	0.09
Age group 2 *N* = 442	48.2 (3.8)	−0.87	−1.65	−0.08
Age group 3 *N* = 442	63.9 (7.7)	−0.23	−0.72	0.26
ODI, %^a^	34.2 (16.4)			
ODI group 1 (<30%) *N* = 558	18.8 (7.4)	−0.06	−0.74	0.63
ODI group 2 (>29%) *N* = 774	45.3 (11.5)	0.37	−0.32	1.06

CI, confidence interval; ODI, Oswestry Disability Index.

a Statistically significant interaction.

## Discussion

Among 1332 patients with LBP, several factors significantly affected the association between LBP and physical activity: age, educational level, body weight, perceived general health, and disability severity. For most of these factors, LBP severity and the intensity of physical activity were negatively associated – the worse was LBP, the less was physical activity. Younger age, higher educational level, lower BMI and optimal perceived general health predicted higher physical activity. Sex, smoking, marital status and occupation did not show significant effects on the association between LBP and physical activity. The severity of disability, measured by the ODI, showed paradoxical effect on the studied relationship – moderate or severe disability was associated with increasing physical activity.

The generalizability of the results might be affected by several limitations. The study was of cross-sectional design, which always weakens any attempt to draw a strong opinion on a causal relationship. All the data were self-reported and, thus, prone to under- or over-estimation [14]. Patients referred to a highly specialized unit of university clinic may significantly differ from those seen for example, in primary health care. For some patients, LBP was not the only or even the main reason for visiting a clinic. The sample represented patients of particular age, socioeconomic and educational groups and, therefore, the results might be different in unsimilar populations.

The observed association between LBP and physical activity is in line with some previous studies [1,2]. On the other hand, the present findings diverged from other previous reports, which have stated that LBP and physical activity might not be associated [4]. The intensity of LBP has previously been found higher in older people with lower educational level [10,11]. In turn, higher physical activity has often been associated with higher educational level, normal weight, younger age, good general health and milder disability [9]. Thus, the characteristics of particular samples, dissimilar on these factors, might explain variance between the present results and previous knowledge.

Patients with moderate or severe disability caused by LBP demonstrated a paradoxical effect on the association between physical activity and LBP. This finding was, however, in line with several previous reports on the absence of expected correlation between LBP or physical activity and reported disability [4,5]. Perhaps, people with severe disability caused by LBP are more motivated to participate in physical activity as part of overall pain management. The exact reasons for this phenomenon are, however, unknown awaiting for further exploration. The present findings should be confirmed by additional research on the subject – especially, longitudinal studies with repeated-measures design are needed.

### Conclusion

Several factors may significantly affect the mostly negative association between LBP severity and physical activity: age, educational level, body weight, perceived general health, and disability severity. Younger age, higher educational level, lower BMI and optimal perceived general health predicted higher overall physical activity. Sex, smoking, marital status and occupation did not show significant effects on the association between LBP and physical activity. The severity of disability showed paradoxical effect on the relationship between LBP and physical activity – severe disability was associated with increase in physical activity.

## Acknowledgements

### Conflicts of interest

There are no conflicts of interest.
